# Redetermination of eveite, Mn_2_AsO_4_(OH), based on single-crystal X-ray diffraction data

**DOI:** 10.1107/S1600536811044266

**Published:** 2011-11-02

**Authors:** Yongbo W. Yang, Ryan A. Stevenson, Alesha M. Siegel, Gordon W. Downs

**Affiliations:** a Department of Chemistry and Biochemistry, University of Arizona, 1306 E. University Blvd, Tucson, Arizona 85721-0041, USA; b Department of Geosciences, University of Arizona, 1040 E. 4th Street, Tucson, Arizona 85721-0077, USA; c Department of Molecular and Cellular Biology, University of Arizona, 1007 E. Lowell Street, Tucson, Arizona 85721-0106, USA; d University High School, 421 N. Arcadia Avenue, Tucson, Arizona 85711-3032, USA

## Abstract

The crystal structure of eveite, ideally Mn_2_(AsO_4_)(OH) [dimanganese(II) arsenate(V) hydroxide], was refined from a single crystal selected from a co-type sample from Långban, Filipstad, Varmland, Sweden. Eveite, dimorphic with sarkinite, is structurally analogous with the important rock-forming mineral andalusite, Al_2_OSiO_4_, and belongs to the libethenite group. Its structure consists of chains of edge-sharing distorted [MnO_4_(OH)_2_] octa­hedra (..2 symmetry) extending parallel to [001]. These chains are cross-linked by isolated AsO_4_ tetra­hedra (..*m* symmetry) through corner-sharing, forming channels in which dimers of edge-sharing [MnO_4_(OH)] trigonal bipyramids (..*m* symmetry) are located. In contrast to the previous refinement from Weissenberg photographic data [Moore & Smyth (1968 ▶). *Am. Mineral.* **53**, 1841–1845], all non-H atoms were refined with anisotropic displacement param­eters and the H atom was located. The distance of the donor and acceptor O atoms involved in hydrogen bonding is in agreement with Raman spectroscopic data. Examination of the Raman spectra for arsenate minerals in the libethenite group reveals that the position of the peak originating from the O—H stretching vibration shifts to lower wavenumbers from eveite, to adamite, zincolivenite, and olivenite.

## Related literature

For background to eveite, see: Moore (1968 ▶); Moore & Smyth (1968 ▶); Hålenius & Westlund (1998 ▶). For other minerals of the libethenite group, see: Hawthorne (1976 ▶); Cordsen (1978 ▶); Toman (1978 ▶); Li *et al.* (2008 ▶). Correlations between O—H streching frequencies and O—H⋯O donor–acceptor distances were given by Libowitzky (1999 ▶).

## Experimental

### 

#### Crystal data

Mn_2_AsO_4_(OH)
*M* *_r_* = 265.81
Orthorhombic, 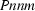

*a* = 8.5478 (16) Å
*b* = 8.7207 (16) Å
*c* = 6.2961 (12) Å
*V* = 469.33 (15) Å^3^
*Z* = 4
Mo *K*α radiation
μ = 12.29 mm^−1^
*T* = 293 K
0.05 × 0.05 × 0.04 mm

#### Data collection

Bruker APEXII CCD area-detector diffractometer
Absorption correction: multi-scan (*SADABS*; Sheldrick, 2005 ▶) *T*_min_ = 0.579, *T*_max_ = 0.639
3315 measured reflections
911 independent reflections
849 reflections with *I* > 2σ(*I*)
*R*_int_ = 0.015

#### Refinement

*R*[*F* ^2^ > 2σ(*F* ^2^)] = 0.021
*wR*(*F* ^2^) = 0.055
*S* = 1.09
911 reflections
49 parameters
All H-atom parameters refined
Δρ_max_ = 1.28 e Å^−3^
Δρ_min_ = −1.15 e Å^−3^

### 

Data collection: *APEX2* (Bruker, 2004 ▶); cell refinement: *SAINT* (Bruker, 2004 ▶); data reduction: *SAINT*; program(s) used to solve structure: *SHELXS97* (Sheldrick, 2008 ▶); program(s) used to refine structure: *SHELXL97* (Sheldrick, 2008 ▶); molecular graphics: *XtalDraw* (Downs & Hall-Wallace, 2003 ▶); software used to prepare material for publication: *publCIF* (Westrip, 2010 ▶).

## Supplementary Material

Crystal structure: contains datablock(s) I, global. DOI: 10.1107/S1600536811044266/wm2546sup1.cif

Structure factors: contains datablock(s) I. DOI: 10.1107/S1600536811044266/wm2546Isup2.hkl

Additional supplementary materials:  crystallographic information; 3D view; checkCIF report

## Figures and Tables

**Table 1:** Hydrogen-bond geometry (Å, °)

*D*—H⋯*A*	*D*—H	H⋯*A*	*D*⋯*A*	*D*—H⋯*A*
O1—H1⋯O4^i^	0.88 (6)	2.57 (4)	2.885 (2)	102 (3)

Symmetry code: (i) 
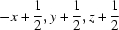
.

**Fig. 1.**
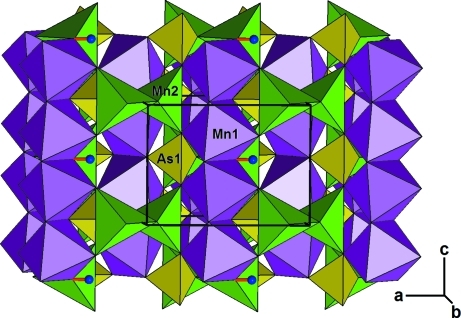
Crystal structure of eveite. Small spheres represent H atoms, which are drawn with arbitrary radii.

**Fig. 2.**
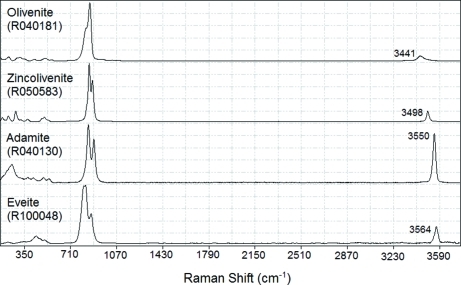
Raman spectra of eveite, adamite, zincolivenite, and olivenite. The spectra are shown with vertical offset for clarity.

## supplementary crystallographic information

### Comment

Eveite, Mn_2_(AsO_4_)(OH), dimorphic with sarkinite, is a rare secondary mineral and belongs to the libethenite group of minerals, which are structurally analogous with the important rock-forming mineral andalusite, Al_2_OSiO_4_. The libethenite group includes seven phosphate and arsenate members: libethenite Cu_2_(PO_4_)(OH), zincolibethenite CuZn(PO_4_)OH, olivenite Cu_2_(AsO_4_)(OH), zincolivenite CuZn(AsO_4_)(OH), auriacusite Fe^3+^Cu^2+^(AsO_4_)O, adamite Zn_2_(AsO_4_)(OH), and eveite. Except monoclinic *P*2_1_/*n* olivenite (Li *et al.*, 2008), all other minerals in this group display orthorhombic symmetry and crystallize in the space group *Pnnm*.

Eveite from Långban, Sweden was first described by Moore (1968) as orthorhombic, with space group *Pnnm*, unit-cell parameters *a* = 8.57 (1), *b* = 8.77 (1), and *c* = 6.27 (1) Å, and an empirical formula (Mn_1.93_Ca_0.07_)_2_(AsO_4_)(OH). Its structure was subsequently determined by Moore & Smyth (1968) using the X-ray intensity data measured from Weissenberg photographs. However, the specimen investigated by Moore & Smyth (1968) was not a single crystal. The eveite crystals they examined were invariably warped and consisted of composites in near-parallel growth. Their structure refinement on the atomic coordinates and isotropic displacement parameters of all non-hydrogen atoms converged with *R* = 15% for 445 observed reflections and *R* = 19% for all 639 reflections. Since then, no further crystallographic study on eveite has been reported. In our efforts to understand the relationships between the hydrogen environments and Raman spectra of hydrous minerals, we concluded that the structural information of eveite needs to be improved. During the course of sample identification for the RRUFF project (http://rruff.info), we found a high-quality single-crystal of eveite from the co-type sample Flink 32/22, and performed a detailed structure refinement.

The structure of eveite is characterized by chains of edge-sharing [Mn1O_4_(OH)_2_] octahedra (..2 symmetry) running parallel to [001]. These chains are cross-linked by sharing corners with isolated AsO_4_ tetrahedra (..*m* symmetry) to form an open framework. Channels in the framework contain dimers of edge-sharing [Mn2O_4_(OH)] trigonal bipyramids (..*m* symmetry), which share corners with the [Mn1O_4_(OH)_2_] octahedra and AsO_4_ tetrahedra (Fig. 1). The average <Mn1—O>, <Mn2—O>, and <As1—O> bond lengths are 2.197, 2.119, and 1.690 Å, respectively, agreeing well with those reported by Moore & Smyth (1968). Nevertheless, the AsO_4_ tetrahedron in our study is much less distorted than reported previously. The respective longest and shortest As—O bond lengths within the AsO_4_ tetrahedron are 1.733 and 1.654 Å from Moore & Smyth (1968), as compared to 1.693 (2) and 1.683 (2) Å in our study.

The hydrogen bonding in eveite is found between O1 and O4, with the former as the H-donor and the latter as the H-acceptor, and the O1—O4 distance of 2.885 (2) Å. Our Raman spectra of eveite (Fig. 2; also see http://rruff.info/eveite) shows a major band at ~3564 cm^-1^ that is attributable to the O—H stretching vibration (ν_O—H_). This peak position is consistent with the refined O—H···O distance according to the correlation between ν_O—H_ and O—H···O distances for minerals (Libowitzky, 1999). The FTIR absorption spectra of eveite from Hålenius & Westlund (1998) also display a sharp band at ~3560 cm^-1^, in concurrence with our Raman spectroscopic measurement. However, the angle O1—H···O4 (102°) appears to be too small for hydrogen bonding. Similar results have been observed in other members of the libethenite group and a bifurcated hydrogen bonding model has been proposed to account for such a hydrogen bonding scheme (Cordsen, 1978).

Examination of the Raman spectra of arsenate minerals in the libethenite group, namely eveite, adamite, olivenite, and zincolivenite, documented in the RRUFF Project (RRUFF deposition #: R100048, R040130, R050583, and R040181, respectively), reveals that the position of the ν_O—H_ band appears to shift significantly to lower wavenumbers from eveite (3564 cm^-1^), to adamite (3550 cm^-1^), zincolivenite (3498 cm^-1^), and olivenite (3441 cm^-1^) (Fig. 2). This observation can be ascribed to the different cations bonded to O1. In the four arsenate minerals mentioned above, each O1 is bonded to three *M* cations (*M* = Cu, Zn, Mn), in addition to the H atom. The average *M*—O distance decreases from 2.127 Å in eveite, to 2.044 Å in adamite (Hawthorne, 1976), 1.987 Å in zincolivenite (Toman, 1978), and 1.982 Å in olivenite (Li *et al.*, 2008), indicating that the bond-valence contributions from the *M* cations to O1 increase in the same order. To compensate this bond valence change in O1, the O1—H distance has to increase from eveite to olivenite, thus resulting in the shift of the ν_O—H_ position to a lower wavenumber.

### Experimental

The eveite crystal used in this study is from a co-type sample donated by William W. Pinch and is in the collection of the RRUFF project (deposition No. R100048; http://rruff.info). The chemical composition analyzed by Moore (1968) was adopted for the structure refinement.

### Refinement

The H atom was located from difference Fourier syntheses and its position and isotropic displacement parameter refined freely. An ideal chemistry was assumed during the refinement, as the overall effects of the trace amount of Ca on the final structure results are negligible. The highest residual peak in the difference Fourier maps was located at (0.6489, 0.1494, 0), 1.24 Å from H, and the deepest hole at (0.1562, 0.2561, 0), 0.74 Å from As1.

### Crystal data

**Table:**

Mn_2_AsO_4_(OH)	*F*(000) = 496
*M**_r_* = 265.81	*D*_x_ = 3.762 Mg m^−^^3^
Orthorhombic, *P**n**n**m*	Mo *K*α radiation, λ = 0.71073 Å
Hall symbol: -P 2 2n	Cell parameters from 2202 reflections
*a* = 8.5478 (16) Å	θ = 4–32°
*b* = 8.7207 (16) Å	µ = 12.29 mm^−^^1^
*c* = 6.2961 (12) Å	*T* = 293 K
*V* = 469.33 (15) Å^3^	Cuboid, pale gray
*Z* = 4	0.05 × 0.05 × 0.04 mm

### Data collection

**Table:**

Bruker APEXII CCD area-detector diffractometer	911 independent reflections
Radiation source: fine-focus sealed tube	849 reflections with *I* > 2σ(*I*)
graphite	*R*_int_ = 0.015
φ and ω scan	θ_max_ = 32.6°, θ_min_ = 4.0°
Absorption correction: multi-scan (*SADABS*; Sheldrick, 2005)	*h* = −12→5
*T*_min_ = 0.579, *T*_max_ = 0.639	*k* = −13→12
3315 measured reflections	*l* = −7→9

### Refinement

**Table:**

Refinement on *F*^2^	Secondary atom site location: difference Fourier map
Least-squares matrix: full	Hydrogen site location: difference Fourier map
*R*[*F*^2^ > 2σ(*F*^2^)] = 0.021	All H-atom parameters refined
*wR*(*F*^2^) = 0.055	*w* = 1/[σ^2^(*F*_o_^2^) + (0.0327*P*)^2^ + 0.4777*P*] where *P* = (*F*_o_^2^ + 2*F*_c_^2^)/3
*S* = 1.09	(Δ/σ)_max_ = 0.005
911 reflections	Δρ_max_ = 1.28 e Å^−^^3^
49 parameters	Δρ_min_ = −1.15 e Å^−^^3^
0 restraints	Extinction correction: *SHELXL97* (Sheldrick, 2008), Fc^*^=kFc[1+0.001xFc^2^λ^3^/sin(2θ)]^-1/4^
Primary atom site location: structure-invariant direct methods	Extinction coefficient: 0.0034 (7)

### Special details

**Table:**

Geometry. All e.s.d.'s (except the e.s.d. in the dihedral angle between two l.s. planes) are estimated using the full covariance matrix. The cell e.s.d.'s are taken into account individually in the estimation of e.s.d.'s in distances, angles and torsion angles; correlations between e.s.d.'s in cell parameters are only used when they are defined by crystal symmetry. An approximate (isotropic) treatment of cell e.s.d.'s is used for estimating e.s.d.'s involving l.s. planes.
Refinement. Refinement of *F*^2^ against ALL reflections. The weighted *R*-factor *wR* and goodness of fit *S* are based on *F*^2^, conventional *R*-factors *R* are based on *F*, with *F* set to zero for negative *F*^2^. The threshold expression of *F*^2^ > σ(*F*^2^) is used only for calculating *R*-factors(gt) *etc*. and is not relevant to the choice of reflections for refinement. *R*-factors based on *F*^2^ are statistically about twice as large as those based on *F*, and *R*- factors based on ALL data will be even larger.

### Fractional atomic coordinates and isotropic or equivalent isotropic displacement parameters (Å^2^)

**Table:**

	*x*	*y*	*z*	*U*_iso_*/*U*_eq_	
Mn1	0.0000	0.0000	0.24719 (6)	0.01052 (10)	
Mn2	0.35839 (4)	0.13367 (4)	0.5000	0.01082 (11)	
As1	0.24335 (3)	0.25667 (3)	0.0000	0.00735 (9)	
O1	0.3874 (2)	0.3726 (2)	0.5000	0.0101 (3)	
O2	0.4144 (2)	0.3545 (2)	0.0000	0.0115 (3)	
O3	0.1033 (2)	0.3922 (2)	0.0000	0.0171 (4)	
O4	0.22306 (16)	0.14338 (15)	0.2163 (2)	0.0131 (3)	
H1	0.287 (7)	0.394 (6)	0.5000	0.042 (14)*	

### Atomic displacement parameters (Å^2^)

**Table:**

	*U*^11^	*U*^22^	*U*^33^	*U*^12^	*U*^13^	*U*^23^
Mn1	0.01271 (19)	0.01066 (19)	0.00818 (18)	0.00186 (12)	0.000	0.000
Mn2	0.01072 (18)	0.00927 (18)	0.01247 (18)	0.00027 (12)	0.000	0.000
As1	0.00733 (13)	0.00605 (14)	0.00868 (14)	−0.00111 (7)	0.000	0.000
O1	0.0105 (7)	0.0079 (8)	0.0119 (8)	−0.0004 (6)	0.000	0.000
O2	0.0097 (8)	0.0141 (9)	0.0106 (8)	−0.0056 (6)	0.000	0.000
O3	0.0099 (8)	0.0084 (8)	0.0331 (12)	0.0015 (6)	0.000	0.000
O4	0.0141 (6)	0.0143 (6)	0.0108 (6)	−0.0049 (5)	−0.0023 (5)	0.0026 (5)

### Geometric parameters (Å, °)

**Table:**

Mn1—O1^i^	2.1406 (13)	Mn2—O4	2.1296 (14)
Mn1—O1^ii^	2.1406 (13)	Mn2—O4^vi^	2.1296 (14)
Mn1—O2^iii^	2.1630 (13)	Mn2—O3^i^	2.131 (2)
Mn1—O2^i^	2.1630 (13)	As1—O3	1.683 (2)
Mn1—O4^iv^	2.2884 (13)	As1—O4^vii^	1.6915 (14)
Mn1—O4	2.2884 (13)	As1—O4	1.6915 (14)
Mn2—O1	2.0984 (19)	As1—O2	1.6929 (18)
Mn2—O3^v^	2.106 (2)		
			
O1^i^—Mn1—O1^ii^	86.72 (7)	O1—Mn2—O4	91.42 (5)
O1^i^—Mn1—O2^iii^	172.52 (7)	O3^v^—Mn2—O4	122.98 (4)
O1^ii^—Mn1—O2^iii^	94.51 (5)	O1—Mn2—O4^vi^	91.42 (5)
O1^i^—Mn1—O2^i^	94.51 (5)	O3^v^—Mn2—O4^vi^	122.98 (4)
O1^ii^—Mn1—O2^i^	172.52 (7)	O4—Mn2—O4^vi^	114.00 (8)
O2^iii^—Mn1—O2^i^	85.24 (7)	O1—Mn2—O3^i^	164.36 (8)
O1^i^—Mn1—O4^iv^	91.67 (6)	O3^v^—Mn2—O3^i^	75.00 (9)
O1^ii^—Mn1—O4^iv^	81.22 (6)	O4—Mn2—O3^i^	97.05 (5)
O2^iii^—Mn1—O4^iv^	95.81 (6)	O4^vi^—Mn2—O3^i^	97.05 (5)
O2^i^—Mn1—O4^iv^	91.36 (6)	O3—As1—O4^vii^	109.72 (6)
O1^i^—Mn1—O4	81.22 (6)	O3—As1—O4	109.72 (6)
O1^ii^—Mn1—O4	91.67 (6)	O4^vii^—As1—O4	107.26 (9)
O2^iii^—Mn1—O4	91.36 (6)	O3—As1—O2	105.09 (10)
O2^i^—Mn1—O4	95.81 (6)	O4^vii^—As1—O2	112.51 (6)
O4^iv^—Mn1—O4	170.25 (7)	O4—As1—O2	112.51 (6)
O1—Mn2—O3^v^	89.37 (8)		

Symmetry codes: (i) −*x*+1/2, *y*−1/2, −*z*+1/2; (ii) *x*−1/2, −*y*+1/2, *z*−1/2; (iii) *x*−1/2, −*y*+1/2, *z*+1/2; (iv) −*x*, −*y*, *z*; (v) *x*+1/2, −*y*+1/2, *z*+1/2; (vi) *x*, *y*, −*z*+1; (vii) *x*, *y*, −*z*.

### Hydrogen-bond geometry (Å, °)

**Table:**

*D*—H···*A*	*D*—H	H···*A*	*D*···*A*	*D*—H···*A*
O1—H1···O4^viii^	0.88 (6)	2.57 (4)	2.885 (2)	102 (3)

Symmetry codes: (viii) −*x*+1/2, *y*+1/2, *z*+1/2.
